# Maintaining proper health records improves machine learning predictions for novel 2019-nCoV

**DOI:** 10.1186/s12911-021-01537-3

**Published:** 2021-05-27

**Authors:** Koffka Khan, Emilie Ramsahai

**Affiliations:** 1 Department of Computing and Information Technology, The University of the West Indies, St. Augustine, Trinidad and Tobago; 2 UWI School of Business & Applied Studies Ltd (UWI-ROYTEC), 136-138 Henry Street, 24105 Port of Spain, Trinidad and Tobago

**Keywords:** 2019-nCoV, Pneumonia, Machine learning, AdaBoost, Bagging, Classifiers, Disease, Death, Prediction

## Abstract

**Background:**

An ongoing outbreak of a novel coronavirus (2019-nCoV) pneumonia continues to affect the whole world including major countries such as China, USA, Italy, France and the United Kingdom. We present outcome (‘recovered’, ‘isolated’ or ‘death’) risk estimates of 2019-nCoV over ‘early’ datasets. A major consideration is the likelihood of death for patients with 2019-nCoV.

**Method:**

Accounting for the impact of the variations in the reporting rate of 2019-nCoV, we used machine learning techniques (AdaBoost, bagging, extra-trees, decision trees and k-nearest neighbour classifiers) on two 2019-nCoV datasets obtained from Kaggle on March 30, 2020. We used ‘country’, ‘age’ and ‘gender’ as features to predict outcome for both datasets. We included the patient’s ‘disease’ history (only present in the second dataset) to predict the outcome for the second dataset.

**Results:**

The use of a patient’s ‘disease’ history improves the prediction of ‘death’ by more than sevenfold. The models ignoring a patent’s ‘disease’ history performed poorly in test predictions.

**Conclusion:**

Our findings indicate the potential of using a patient’s ‘disease’ history as part of the feature set in machine learning techniques to improve 2019-nCoV predictions. This development can have a positive effect on predictive patient treatment and can result in easing currently overburdened healthcare systems worldwide, especially with the increasing prevalence of second and third wave re-infections in some countries.

## Background

A zoonotic coronavirus has crossed species to infect human populations. This virus, unofficially known as 2019-nCoV, was first detected in people exposed to a seafood or wet market in Wuhan, China. Similar to other pathogenic human respiratory coronaviruses, 2019-nCoV triggers respiratory disorders that are severe in some cases. More than 1,133,758 confirmed cases were registered as of 5 April 2020, with 62,784 deaths [1].

This disease has now evolved to be spread by human-to-human communication. Typical clinical signs in 2019-nCoV patients include fatigue, dry cough, trouble swallowing (dyspnoea), headache, and pneumonia. The development of the disease can result in progressive respiratory failure due to alveolar damage (as observed from computerized transverse chest tomography images) and even death.

As a ribonucleic acid (RNA) virus [2], 2019-nCoV also has an intrinsic characteristic of a high mutation rate; however, as found in other coronaviruses, the mutation rate may be significantly lower than those of other RNA viruses owing to its genome-encoded exonuclease. This feature offers the potential for this recently developed zoonotic viral pathogen to evolve and more easily spread from person to person, likely becoming more virulent.

Recently, machine learning techniques have been applied successfully to a wide range of problems including those in the health care field [3–5]. Since the appearance of 2019-nCoV, many researchers have employed machine learning techniques to predict patterns related to various genotypic and phenotypic viral traits combined with human social behaviour. Randhawa et. al. introduced an intrinsic genomic signature of the COVID-19 virus and used it for an ultra-fast, scalable, and highly accurate classification of entire 2019-nCoV virus genomes along with a machine learning-based alignment-free approach [6]. Ozturk et al. proposed a deep model to use X-ray images for early detection of COVID-19 cases [7]. They obtained a 98.08% accuracy and 87.02% accuracy for discrete and multi-classes. Their DarkCovidNet model can help clinicians make the diagnosis more rapidly and accurately. In [8], the problem of automatic classification of pulmonary diseases including the recently emerged 2019-nCoV from X-ray images was considered. A state-of-the-art convolutional neural network (CNN) called Mobile Net was trained from scratch to investigate the importance of the extracted features for the classification task. A classification accuracy of 87.66% was achieved among seven classes. This approach also achieved 99.18%, 97.36% sensitivity and 99.42% specificity in 2019-nCoV identification.

Researchers have also used artificial intelligence (AI) algorithms to combine chest CT findings with clinical symptoms, exposure history and laboratory testing to quickly diagnose patients who are 2019-nCoV positive [9]. Their system correctly identified 17 of 25 (68%) patients and achieved an area under the curve of 0.92. In [10], a clinical text classification paradigm using weak supervision and deep representation was proposed to reduce human effort. Support vector machine (SVM), random forest (RF), multilayer perceptron neural network (MLPNN), and CNN were tested using a weak supervision paradigm. Precision, recall, and F1 score were used as the metrics to evaluate the performance. CNN achieved the best performance. Although many other studies focusing on supervised machine learning applied to 2019-nCoV in various ways have been reported in the literature, no studies have explored predictions of death from 2019-nCoV as we start to explore in this paper. These early experiments have met with varying success. However, with the introduction of new datasets, researchers are eager to engage various machine learning techniques to help manage this outbreak.

Initial datasets were very sparse and at first included only a single country. Consequently, as 2019-nCoV spread, the increased awareness and recordkeeping meant that the datasets grew with respect to the number of features and size. Nonetheless, at the beginning of April 2020, only a small number of datasets were available to researchers. Nevertheless, we choose two datasets from Kaggle [11, 12]. Table 1 shows the recovered, confirmed cases and deaths due to this virus over time.

**Table 1:** Recovered, confirmed cases and deaths by 2019-nCoV virus over time [11, 12]

Date	Deaths		Confirmed		Recovered	
	China	Korea	China	Korea	China	Korea
1/22/2020	17	0	548	1	28	0
1/25/2020	42	0	1406	2	39	0
1/28/2020	131	0	5509	4	101	0
1/31/2020	213	0	9802	11	214	0
2/1/2020	259	0	11,891	12	275	0
2/5/2020	563	0	27,440	18	1115	1
2/9/2020	905	0	39,829	27	3219	3
2/13/2020	1369	0	59,895	28	6217	7
2/17/2020	1864	0	72,434	30	12,462	10
2/21/2020	2238	2	75,550	204	18,704	17
2/25/2020	2665	12	77,754	977	27,676	24
2/29/2020	2837	17	79,356	3150	39,320	28
3/1/2020	2872	18	79,932	3736	42,162	30
3/5/2020	3015	35	80,537	5766	52,292	88
3/9/2020	3123	51	80,860	7382	58,804	166
3/13/2020	3180	67	80,945	7979	64,196	510
3/17/2020	3230	81	81,058	8320	68,798	1401
3/21/2020	3259	102	81,305	8799	71,857	2612
3/23/2020	3274	111	81,439	8961	72,814	3166

We focused our work on predicting impending death from 2019-nCoV based on the given data. Our aim was to develop a tool for precise risk prediction to facilitate urgent treatment targeted at high-risk individuals. Our analysis focuses on many state-of-the-art algorithmic developments that have demonstrated promise in improving disease prediction. The development of a more in-depth understanding and theoretical study of critical problems related to algorithmic construction and learning theory was crucial in the advance of these methods. These include trade-offs for optimizing efficiency [13] using physically reasonable constraints, and integration of prior information and uncertainty. Our contributions are as follows:

1. We created, trained and tested models based on five machine learning techniques from the two Kaggle datasets. Machine learning hyper-parameters were tuned to obtain models with optimal performance.
2. We confirmed that using the patient’s ‘disease’ history resulted in more than a sevenfold increase in the accuracy of prediction of death.
3. We developed a machine learning tool for death prediction to facilitate urgent treatment targeted at high-risk individuals. The tool works for ‘early’ datasets with few deaths but will improve with the addition of more patient cases. Thus, it can be used for countries now developing cases and those with many cases.
4. In the future, improved death predictions can assist worldwide healthcare systems in fighting this outbreak.

The rest of this paper is organized as follows. In “Related work” section describes important work, concepts and metrics associated with our work. We discuss different machine learning techniques and statistical metrics used in this paper. We then outline the method used to set up our experiments including dataset descriptions and parameters utilized for machine learning techniques in “Methods” section. Our results are given in “Results” section together with a discussion giving the importance of using a patient’s ‘disease’ history as a feature in the 2019nCoV datasets. In “Discussion” section, we first analyse our results and then discuss multi-class AUC generalizations, ROC curves and hyper-parameter settings in depth. Finally, we present our conclusions in “Conclusion” section.

## Related work

Here we provide a brief explanation of the three ensemble and two conventional methods used in machine learning. These are important because they are used to build the models used for predictions in our experiments. Then, we discuss the metrics used to evaluate the performance of these models.

### Ensemble methods

An ensemble is a composite model combining a set of low-performing classifiers to construct an improved classifier. An individual vote is performed by the classifier and the final prediction label is returned, resulting in majority voting [14]. In essence, ensemble learning methods are meta-algorithms incorporating many methods of machine learning into one predictive model to improve performance. We selected three ensemble methods based on the literature performance on assisting with pandemic predictions [15–17]. These are AdaBoost, bagging and extra-trees classifiers. These methods are described below.

#### AdaBoost classifier

AdaBoost or adaptive boosting combines several classifiers to improve classifier accuracy. AdaBoost is an iterative ensemble method. It creates a strong classifier by combining several poorly performing classifiers to obtain effective classifier with high precision [18]. The basic idea behind Adaboost is to set classifier weights and train the data sample in each iteration to ensure precise predictions of unusual observations [19]. Many other machine learning techniques can be used as base classifier if they accept weights on the training set [20].

Suppose we are given a set of training data (*x*_1_*, y*_1_),…,(*x*_*n*_*, y*_*n*_) where the output variable is *y*_*i *_= 1, 2,…,*M*. *M* is the number of classes. The goal is to find a classification rule *Y*(*x*) from the training data, for example, the rule with the lowest misclassification error rate. Thus, when given a new input *x*_*i*_, we can assign it a class label *y* from 1,…,*M*. AdaBoost constructs a classifier starting with the unweighted training sample. The classifier is used to produce class labels. The weight of the training data point is increased (boosted) if a training data point is misclassified. Then, using the new weights, which are no longer identical, a second classifier is constructed. Then, the weights are increased by misclassified training data, and the process is repeated. Usually, 500 or 1000 classifiers can be set up in this fashion. Each classifier is assigned a value, and the final classifier is specified as the linear combination of each stage’s classifiers. We let *W*(*x*) denote a weak multi-class classifier that assigns a class label to *x*.

We now describe AdaBoost-SAMME, which is used in multi-class settings. The following steps are taken by the algorithm. Initially, Adaboost selects a training subset randomly. The observation weights $w_{i} = \frac{1}{n}, i = 1, 2, \ldots , n$ are initialized. The AdaBoost machine learning model is iteratively trained by selecting the training set based on the accurate prediction of the last training as follows: for each iteration k = 1 to K, (1) fit the classifier *W*^*k*^(*x*) to the training data using *w*_*i*_. (2) Calculate the error rate by assigning the weight to the trained classifier according to the accuracy of the classifier: $err^{k} = \mathop \sum \nolimits_{i = 1}^{n} w_{i} \left( {c_{i} \ne Y^{\left( k \right)} \left( {x_{i} } \right)} \right)/\mathop \sum \nolimits_{i = 1}^{n} w_{i} .$ A more accurate classifier will get a higher weight. (3) Calculate the weight of the weak classifier according to $\alpha^{\left( k \right)} = \log (1 - err^{\left( k \right)} /err^{\left( k \right)} ) + \log \left( {M - 1} \right)$. For our 3-class system, we need only $err^{\left( k \right)} < 2/3$. (4) Update the weights by assigning the higher weight to wrongly classified observations so that in the next iteration these observations will obtain the higher probability for classification: *w*_*i *_= *w*_*i*_*.exp*(*α*^(*k*)^*.*(*c*_*i*_ 6 ≠ *Y* ^(*k*)^(*x*_*i*_)) where *i* = 1*, *2*,*…*,n*. (v) Re-normalize *w*_*i*_. In the final step, output an approximation to the Bayes classifier by performing a “vote” across all of the learning algorithms: $C^{*} \left( x \right) = \mathop \sum \nolimits_{k = 1}^{K} \alpha^{\left( k \right)} .Y^{\left( k \right)} \left( {x_{i} } \right) = m$.

#### Bagging classifier

A bagging (decision tree) classifier is a meta-estimator ensemble that trains base classifiers on the original dataset’s random subsets with different subset used for each base classifier and then aggregates their individual predictions (either by voting or by averaging) to form a final prediction [21]. A learning set *S* consists of data (*x*_*n*_*,y*_*n*_)*,n* = 1,…,*N* where *x* are either class labels (‘gender’ and ‘disease’) or a numerical response (‘age’). Assume that we can use this learning set to generate a predictor *ν*(*x,S*). Now, suppose that a sequence of learning sets *S*_*j*_ are given, each consisting of independent observations from the same underlying distribution as *S*. Our task is to use *S*_*j*_ to obtain a better predictor than the single predictor *ν*(*x,S*) of the learning set. The constraint is imposed that only the sequence of predictors *ν*(*x,S*) is permitted. A training set of size N is sampled for each trial t = 1, 2, …T with substitution from the original instances. This training set is the same size as the original data set but may not include any instances, while others appear more than once. An obvious procedure is to replace *ν*(*x,S*) with an average over *j*, if *y* is numerical that is *ν*_*agg*_(*x*) = *E*_*S*_(*x, S*) where the subscript *agg* is the aggregation function and *E*_*S*_ is the expectation over *S*. If a class *k* ∈ 1, 2, …,*K* is predicted, then it could be aggregated by voting. This is performed by taking the *k* for which *N*_*k*_ is maximum. However, typically we have a single learning set *S*. Nevertheless, a process leading to *ν*_*agg*_ can still be accomplished by taking repeated bootstrap samples *S*^(*T*)^ from *S* forming *ν*(*x, S*^(*T*)^). Thus, a sample classifier is created by the learning system, and the final classifier is produced by combining the classifiers from these trials. If *y* is numerical, *ν*_*T*_ becomes *ν*_*T*_(*x*) = *avg*_*T*_*ν*(*x, S*^(*T*)^) where *avg* is the average function. If *y* is a class label, *ν*(*x, S*^(*T*)^) becomes a vote for *ν*_*T*_(*x*).

Suppose a patient requires a 2019-nCoV symptom-based diagnosis. The patient would prefer to do multiple tests with many doctors rather than using only one doctor. The most common diagnosis is expected to be the correct diagnosis and a consensus decision from a wide number of doctors is expected more accurate. In bagging, each doctor will act as a version of a particular predictor. An ensemble is created by having multiple versions of a predictor. We note this can be created by one or more doctors. To train each predictor, bagging creates a training data set or bootstrap sample. Usually, a bagging meta-estimator can be used as a means for reducing the variance of a black-box estimator (e.g., a decision tree), integrating randomization into its construction process and then making an ensemble of the results [22]. We choose the decision tree as the sub-classifier of the bagging algorithm. The underlying concept of bagging is that variation is minimized by averaging models and the accuracy of “weak” classifiers is increased. “Weak” classifiers are classified as classifiers that alter their final predictions drastically with no modifications to training data. In bagging, we repeatedly sample from a training set using simple random sampling with replacement. A single “weak” classifier is trained for each bootstrap sample. Then, these classifiers are used on test data to predict class labels and the class that receives the majority of the votes wins (ties are resolved arbitrarily).

#### Extra-trees classifier

This class implements a meta-estimator that fits a number of randomized decision trees (i.e. extra-trees) on different dataset sub-samples and uses the average to improve the predictive accuracy and balance over-fitting power [23]. The algorithm for extra-trees generates an ensemble of unpruned decision trees. By picking cut-points entirely at random, it separates the nodes and uses the whole learning sample *S* (rather than a bootstrap replica) to expand the trees. The algorithm first selects an input variable to divide the data at each stage of the tree expansion *u*_1_,…*,u*_*K*_. Then, it performs *K* splits to produce pieces *p*_1_,…,*p*_*K*_. This is done by determining a random cut-point *u*_*c*_ uniformly in [*u*^*S*^_*min*_*, u*^*S*^_*max*_]*,* where *u*^*S*^_*max*_ and *u*^*S*^_*min*_ are the maximal and minimal values of *u* in the data sample *S*, respectively. If the input variable is continuous, then the cut-point is also chosen randomly, i.e. independently of the class labels [24]. The algorithm returns a split *s*_*ζ*_ such that it is maximum in *S* = *s*_*i*_*,S* where *i* = 1,…,*K*. It has two parameters: *K*, which is the number of randomly chosen input variables for each node, and *n*_*min*_, which is the minimum sampling size for node splitting. With the complete learning sample, these parameters are used in many iterations to create an ensemble model. To yield the final prediction, the predictions of the trees are aggregated by casting a vote and obtaining the majority. The underlying rationale of extra-trees is that the precise randomization of the cut-point and input variable paired with the average of the ensemble should be able to minimize variation more strongly than the weaker randomization strategies.

### Conventional methods

Many conventional learning algorithms have attracted intense attention in many research fields [25]. We selected two conventional methods based on their performance for assisting with pandemic predictions as reported in the literature[26]. These methods are the decision tree and k-nearest neighbour (k-NN) classifiers.

They are described below.

#### Decision tree classifier

Prior to describing in detail the construction of this classifier, we define *D* as the data set. *D* is built by *m* attributes and *n* records. *x*_1_*, x*_2_,…,*x*_*i*_. The target variable is *y*_*i *_= {0*,*1} where 1 ≤ *i* ≥ *n*. Therefore, a record can be expressed as *x*_*i *_= [*x*_*i*1_*, x*_*i*2_,…,*x*_*im*_*,y*_*i*_] and *D* = {*x*_*i*_|1 ≤ *i* ≥ *n*}.

Decision tree algorithms [27–30] classify records by conjunctive rules (e.g. ‘disease’ = yes and ‘sex’ = female and ‘age’ ≥ 60). Several decision tree algorithms apply information theory to separate data by computing the entropy iteratively. When the data is split on the basis of attribute *a*, we denote this entropy by *H*(*D*) and the information gain by *IG*(*D,a*). The expected value of the contained information or entropy is given by *H*(*D*) = Σ*p*(*b*)*log*(*b*), where *D* is the training data set, *Y* is the target variable in *D*, *b* is a classified value in *Y*, and *p*(*b*) is the probability that an object in *D* is classified as *b*.

The amount of uncertainty reduced due to the split is the information gain, which is given by *IG*(*D,a*) = *H*(*T*) − Σ*p*(*a*)*H*(*a*), where *A* is an attribute based on the split, *p*(*a*) is the probability that an object in *D* contains attribute *A* = *a*, and *H*(*a*) is the entropy of the subset of D, where attribute *A* = *a*. The decision tree chooses the attribute with the greatest information gain as a splitting criterion at a local level. The decision tree algorithm chooses the attribute with the highest information gain to be a node after determining the information gain of each attribute, which splits the data set into two or more subsets. The procedure continues iteratively until a complete decision tree is constructed.

#### k-nearest neighbor classifier

The k-nearest neighbor (k-NN) classifier [31, 32] calculates the class membership of a test patient sample by using the k closest neighbors in an outcome of a majority vote. Provided a patient with a death outcome to be imputed and a pool of other patients with ‘similar’ features, in terms of disease similarity, the algorithm searches for the k-closest subjects and infers an estimation for the required value outcome. Initially, the distance from the current patient and the other candidate subjects is computed. A weighted average of the respective values is then obtained in the k of the most related patients and used as a plausible estimate of the required patient. The process is iterated for each outcome value of the given patients to impute the whole dataset. The algorithm takes into account the disease feature in the patient data and controls both the mixed existence of the feature data with the inclusion of these in the distance estimation of multiple classes (‘recovered’, ‘isolated’ and’ death’). The class information is maintained for each of the nearest k neighbors. If there are more than two winners in the majority vote, then there is a tie, which is arbitrarily broken to determine the winner.

The k-NN algorithm proceeds as follows for an *i*th subject with a death outcome value to be imputed. To account for the disparity between the ranges, the features of the subject sample, along with its candidate samples, are normalized to the [1, 1] interval. The difference between subject *i* and each candidate *j* is then determined by applying the Minkowski metric. Let *v* = (*v*_1_*, v*_2_,…,*v*_*N*_) and *u* = (*u*_1_*, u*_2_,…,*u*_*N*_) be the feature vectors of subject *i* and candidate *j*, respectively. The distance between *v* and *u* is given by $\left( {\mathop \sum \nolimits_{i = 1}^{N} \left| {v_{i} - u_{i} } \right|^{p} } \right)^{1/p}$ where *p* is an integer between *v*_*i*_ and *u*_*i*_. If either *v*_*i*_ or *u*_*i*_ is absent, or both, the feature of the *i*th index does not add to the distance. Once the distances to all the candidates have been determined, the *k* closest ones are chosen.

### Statistical metrics

Once a model based on machine learning techniques is constructed, it is necessary to evaluate its performance [33]. The desired model to assist in providing the necessary treatment for the patients that most at risk must be reliable at predicting deaths. We use the accuracy, precision, recall and F1-Score statistical metrics to measure the performance of the models in our experiments [34, 35]. To calculate these values, several other values are necessary. True positive (TP), True negative (TN), False negative (FN) and False positive (FP). Both TP and TN indicate a consistent result between the prediction and the actual outcome. Conversely, FN and FP indicate that the predictions are not the same as the actual condition. For our death prediction, we recognized that FP results are not as dangerous as FN. Therefore, our aim was to minimize the number of false negatives (FN) because these are the cases where death is not correctly predicted and the patient does not receive adequate medical attention. We briefly describe these metrics and their calculations in the following sub-sections.

#### Accuracy

The most popular classification metric is accuracy, defined as the fraction of the samples correctly predicted. It is described by Eq. 1.

1 $$Accuracy = \frac{TP + TN}{{TP + TN + FP + FN}}$$

#### Precision

Precision is the proportion of the successfully predicted occurrences that are in fact positive. It is described by Eq. 2.

2 $$Precision = \frac{TP}{{TP + FP}}$$

#### Recall

Recall (also called sensitivity) is the proportion of successful events that are predicted correctly. It is described by Eq. 3.

3 $$Recall = \frac{TP}{{TP + FN}}$$

#### F1-Score

The F1-score is the harmonic mean of recall and precision, with the greater score interpreted as a better model. It is described by Eq. 4.

4 $$F1 - Score = \frac{2*(Precision*Recall)}{{Precision + Recall}}$$

## Methods

The overall steps used in our study are outlined in Fig. 2. In step 1, we obtained input data. We used two datasets from Kaggle [11, 12]. These datasets were obtained on March 30 2020. Dataset1 has 1086 cases with nineteen (19) features and dataset2 2756 cases with eighteen (18) features. The number of ‘death’ patients from dataset1 was 63, ‘released’ was 314 and ‘isolated’ was 709. The number of ‘death’ patients from dataset2 was 53, ‘released’ was 874 and ‘isolated’ was 1828. Training data for Dataset1 consisted of 652 records, while Test data had 164 records. Training data for Dataset2 consisted of 2204 records, while Test data had 552 records.

The feature set and outcome variable were separated and formatted in step 2. Steps 3, 4 and 5 include splitting the dataset into Training and Testing and using the Training data to train and create an appropriate model. Initial experiments predicted two outcomes (‘Alive’ and ‘Death’) and were followed by experiments that predicted three outcomes (‘recovered’, ‘isolated’ or ‘death’). The common outcome is ‘death’. We aim to develop a model that accurately predicts ‘death’. Hence we construct a model for ‘death’ independently. We build a model for predicting the probability of death that would not be used to predict ‘recovered’ or ‘isolated’. However, this model may not be optimal. Further, at steps 6 and 7, we tune the model hyper-parameters to obtain optimal results. During step 8, we use our Test data and optimal model to obtain 2019-nCoV predictions. Finally, in step 9, we notify relevant authorities of the outcome.

The models were evaluated using accuracy, precision, recall and F1-score. For each patient, we predict each outcome using a number of features. The initial features were ‘country’, ‘gender’ and ‘age’ from both datasets yielding three sub-samples. We filtered out patient cases that do not include all of the features. This created a total number of 816 cases in dataset1 and 2754 cases in dataset2. Then, we included the patient’s disease history feature, ’disease’ from dataset2, producing our final sub-sample. There were no invalid cases for this feature set. We created an outcome variable for the categorical outcome of ‘recovered’, ‘isolated’ and ‘death’. The following lists shows our four sub-samples.

- The first sub-sample was obtained from dataset1 and has the feature set ‘country’, ‘age’ and ‘sex’ with two outcomes (‘alive’ and ‘death’).
- The second sub-sample was obtained from dataset1 and has the feature set ‘country’, ‘age’ and ‘sex’ with three outcomes (‘recovered’, ‘isolated’ or ‘death’).
- The third sub-sample was obtained from dataset2 and has the feature set ‘country’, ‘age’ and ‘sex’ with three outcomes (‘recovered’, ‘isolated’ or ‘death’).
- The fourth sub-sample was obtained from dataset2 and has the feature set ‘disease’, ‘age’ and ‘sex’ with three outcomes (‘recovered’, ‘isolated’ or ‘death’).

The models were trained on these four sub-samples using three ensemble and two conventional algorithms. Python version 3.5 and Scikit learn machine learning libraries were used [36]. The models are calibrated or tuned by changing the values of their respective hyper-parameters; for example, the AdaBoost classifier used a decision tree with a maximum depth of 2, learning rate of 2 and number of estimators equal to 100. We take each of the hyper-parameters and vary them randomly within a range using a random number generator within a program loop. For example, the number of estimators varied between 1 and 180. This means that for each experiment, the value for this hyper-parameter can be any value in [1, 1]. Since we seek to construct models with higher Recall values, this was our primary criteria for selecting ‘better’ models. The five machine learning techniques were used with the following hyper-parameter settings in all of the experiments after tuning for optimal performance, see Table 2: The models were trained on 80% of the subsamples and tested on 20%. The following steps were used for each experiment:

1. The data files were retrieved from the input directory.
2. The data were cleaned.
3. The outcome variable was defined.
4. The data were divided into training and testing sets.
5. Non-numeric features were mapped to numeric values.
6. The machine learning technique and hyper-parameters were chosen.
7. The model was created using the training data.
8. Predictions were obtained by applying the model on the testing set.
9. Evaluations of model performance were performed using the relevant test metrics.

**Table 2:** Optimum hyper-parameter settings for experiments

Setting	AdaBoost	Bagging	Extra-Trees	Decision Tree	k-NN
Base Estimator	None	None	NA	NA	NA
# Estimators	100	10	100	NA	NA
Learning rate	2	NA	NA	NA	NA
Algorithm	SAMME.R	Bagging	Gini	Gini	KDTree
Metric	Mean label accuracy	Mean label accuracy	Gini Impurity	Gini Impurity	Euclidean distance
Random state	None	Random generation	None	Random generation	NA
Max. samples to train needed to train base estimator	NA	1	NA	NA	NA
Out-of-bag samples to estimate generalization error	NA	None	None	NA	NA
Use whole ensemble to fit	NA	Yes	Yes	NA	NA
# Jobs to run in parallel	NA	1	1	NA	1
Random resampling	NA	3141	12	NA	NA
Min. sample to be a leaf	NA	NA	2	2	NA
Sample weighting	NA	NA	All equal, weight of 1	All equal, weight of 1	NA
# of features for best split	NA	NA	Square root of the # of *features*	Max. features = # of features	NA
Min. number of leaf nodes	NA	NA	Unlimited	NA	NA
Split criteria	NA	NA	Impurity level > 0	NA	NA
Reuse previous call to fit and add more estimators to ensemble	NA	No	Yes	NA	NA
Number of neighbours	NA	NA	NA	NA	1

We performed experiments selecting the eventual best model as described in “Results” section. However, once a healthcare system has obtained the highest performing model, they can run the above steps using the model, which provides guidance on how to facilitate the treatment of certain patients based on their health status. For instance, if the health status is death, then measures can be taken to improve the care for the patient. This will enable a more effective use of healthcare resources in the health center or hospital.

## Results

Dataset1 [11] provides daily level details (time series data) from 2019-nCoV on the number of infected cases, deaths and recovery. The data were made available from 22 Jan 2020. The main file that we utilized in this dataset is covid 19 data.csv, which is described by the following:

- Sno—Serial number
- Observation Date—Date of the observation in MM/DD/YYYY
- Province/State—Province or state of the observation (Could be empty when missing)
- Country/Region—Country of observation
- Last Update—Time in Coordinated Universal Time (UTC) at which the row is updated for the given province or country. (Not standardized and so please clean before using it)
- Confirmed—Cumulative number of confirmed cases till that date
- Deaths—Cumulative number of of deaths till that date
- Recovered—Cumulative number of recovered cases till that date

Dataset2 [12] is generated by the KCDC (Korea Centers for Disease Control & Prevention), which announces the information of COVID-19 quickly and transparently. The data were made available from 24 Feb 2020. The main file that we utilized in this dataset is PatientInfo.csv, which contains the following fields: patient id, global num, sex, birth year, age, country, province, city, disease, infection case, infection order, infected by, contact number, symptom onset date, confirmed date, released date, death date and state.

**Fig. 1:**
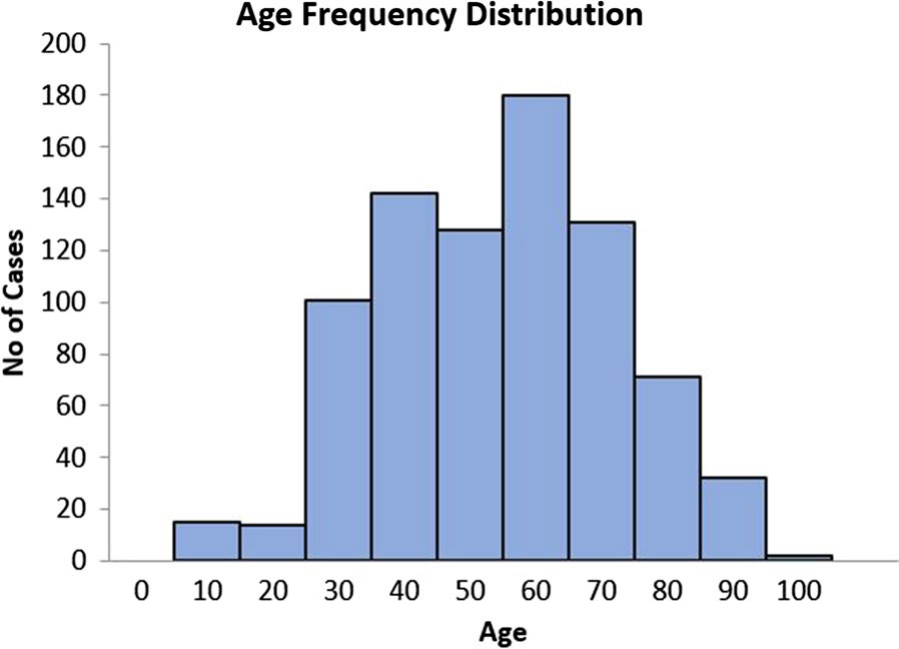
Distribution of patient age for dataset1. Age frequency histogram plot for dataset1

**Fig. 2:**
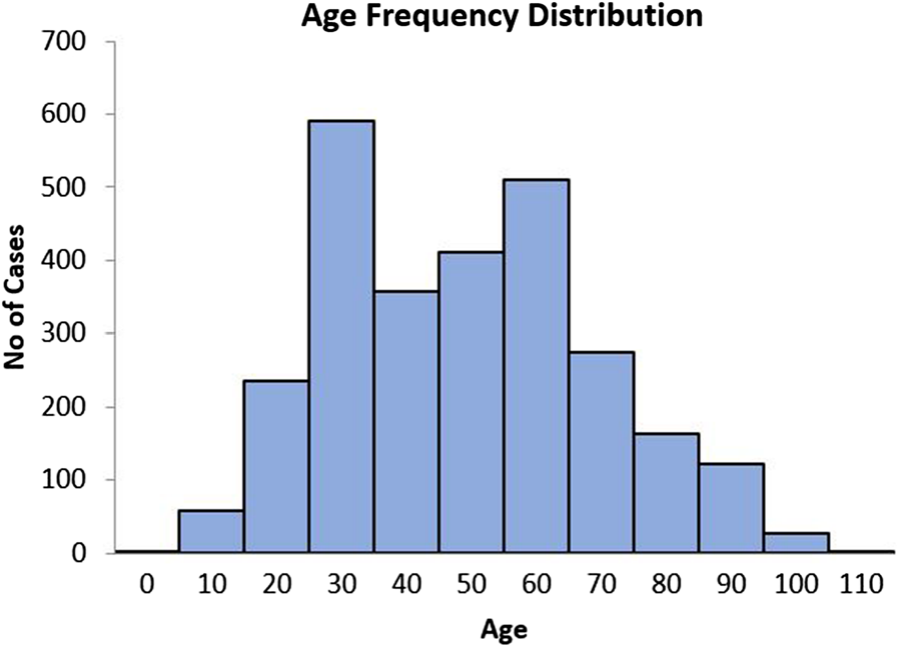
Distribution of patient age for dataset2. Age frequency histogram plot for dataset2

Dataset1 has 42.40% female and 57.6% male patients, while dataset2 has 55.95% female and 44.05% male patients. Neither dataset1 nor dataset2 were skewed based on their age frequency as shown in the age frequency distribution histogram plots on Figs. 1 and 2. However, a further inspection of both datasets shows that it is particularly unbalanced for the outcome of death. There are only 7.10% of deaths in dataset1 and less than 2.0% in dataset2.

We initially tested fourteen classifiers: Gaussian naive Bayes, support vector machine, linear discriminant analysis, one versus rest, gradient boosting, random forest, bagging using a decision tree base estimator, bagging using a logistic regression base estimator, neural network multilayer perceptron, adaboost, bagging, extra-trees, decision tree and k-NN. However, for brevity we select the top five of these models that are described in “Related work” section.

**Fig. 3:**
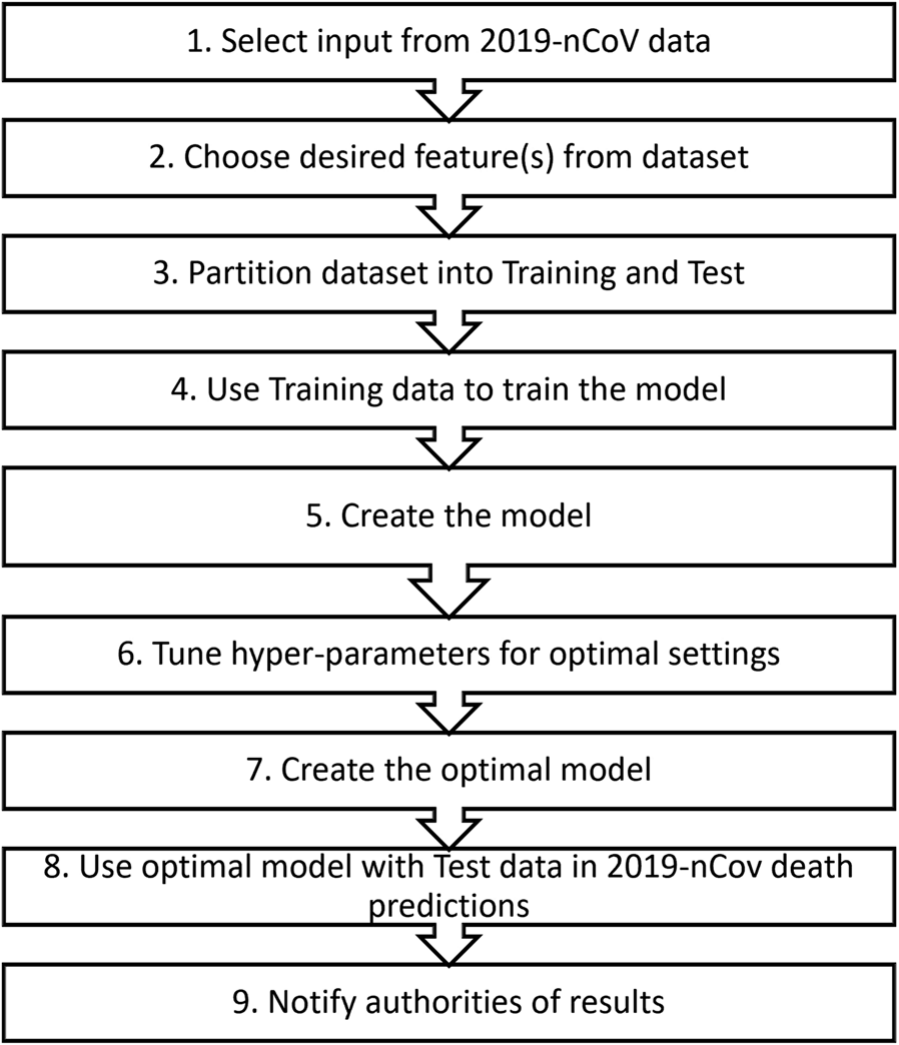
Major steps outlined in our method

Training models on unbalanced data produce inaccurate findings for the prediction on death. This is due to the vast number of alive cases. Our initial tests indicate high (0.94–0.97) precision, recall and F1-scores for survival prediction (alive) yet very low (0.31–0.50) for death prediction as shown in Table 3 for the model trained on sub-sample one. The very low recall values (0.31–0.38) are attributed to the large number of incorrect predictions for deaths (FN). Improving theses death predictions facilitates targeted treatment of high-risk patients. Given that predicting deaths is preferable to having high model accuracy (0.6–0.91), obtaining a high recall is more significant. Thus, the aim of the subsequent experiments is to obtain a high recall value in the prediction of death. Low accuracy can contribute to low precision and recall when estimating positive data points. Low recall is based on a large number of false negatives (FN) and small number of true positives (TP) (Fig. 3).

**Table 3:** Metrics of machine learning models for two most common outcomes on dataset1

Outcome	Metric	AdaBoost	Bagging	Extra-Trees	Decision Tree	k-NN
Alive	Precision	0.95	0.95	0.94	0.95	0.95
	Recall	0.96	0.97	0.97	0.97	0.95
	F1-Score	0.95	0.96	0.95	0.96	0.95
Death	Precision	0.45	0.50	0.44	0.50	0.42
	Recall	0.38	0.38	0.31	0.38	0.38
	F1-Score	0.42	0.43	0.36	0.43	0.40
	Accuracy	0.60	0.92	0.91	0.91	0.91

Upon separating the outcome into the three categories ‘recovered’, ‘isolated’ and ‘death’, no improvement was obtained in the prediction of death as shown in Table 4. In this experiment, recall remained low (0.31–0.38) for death prediction. Thus, we choose a new dataset with which to build new models. This third experiment was run using sub-sample three as shown in Table 5. However, the recall in predicting ‘death’ was again poor (0.10–0.40). In this experiment precision, recall and F1-score remained low (0.02–0.40) for death prediction. We now introduce ‘disease’ as a feature in the prediction model using sub-sample four, see Table 6. We observed a vast improvement in recall predicting ‘death’ (0.43–0.86). AdaBoost achieved the highest recall value of 0.86, which was slightly better than that of the bagging, extra-tree and decision tree classifiers, all of which scored 0.71. Even though bagging did not achieve the highest recall value for deaths, its overall death prediction was the best with precision, recall and F1-score at 0.71. Additionally, bagging successfully predicted ‘isolated’ cases at a precision (0.72), recall (0.84) and F1-score (0.77).’Isolated’ and ‘death’ prediction facilitates urgent treatment targeted at high-risk individuals. This model minimizes the number of false negatives (FNs) in death predictions so that the patients that require adequate medical attention are accurately identified. It must be noted that even though the k-NN recall value was the lowest (0.43), it improved by more than a threefold (0.43) over its previous performance when ‘disease’ was not part of the model.

**Table 4:** Metrics of machine learning models for three most common outcomes on dataset1

Outcome	Metric	AdaBoost	Bagging	Extra-trees	Decision tree	k-NN
Recovered	Precision	0.29	0.44	0.47	0.38	0.34
	Recall	0.81	0.59	0.56	0.56	0.41
	F1-Score	0.23	0.51	0.51	0.45	0.37
Isolated	Precision	0.82	0.85	0.84	0.84	0.81
	Recall	0.30	0.81	0.83	0.78	0.78
	F1-Score	0.44	0.83	0.83	0.81	0.80
Death	Precision	0.09	0.50	0.44	0.50	0.42
	Recall	0.31	0.38	0.31	0.38	0.38
	F1-Score	0.14	0.43	0.36	0.43	0.40
	Accuracy	0.38	0.74	0.74	0.71	0.69

**Table 5:** Metrics of machine learning models for three most common outcomes on dataset2

Outcome	Metric	AdaBoost	Bagging	Extra-trees	Decision tree	k-NN
Recovered	Precision	0.34	0.40	0.39	0.39	0.29
	Recall	0.12	0.18	0.12	0.12	0.31
	F1-Score	0.18	0.25	0.19	0.19	0.30
Isolated	Precision	0.62	0.69	0.69	0.69	0.66
	Recall	0.50	0.88	0.91	0.91	0.64
	F1-Score	0.55	0.77	0.78	0.78	0.65
Death	Precision	0.02	0.33	0.33	0.33	0.11
	Recall	0.40	0.10	0.20	0.20	0.10
	F1-Score	0.04	0.15	0.25	0.25	0.11
	Accuracy	0.38	0.65	0.65	0.65	0.53

**Table 6:** Metrics of machine learning models for two most common and ‘disease’ outcomes on dataset2

Outcome	Metric	AdaBoost	Bagging	Extra-trees	Decision tree	k-NN
Recovered	Precision	0.22	0.36	0.30	0.30	0.31
	Recall	0.20	0.22	0.11	0.11	0.39
	F1-Score	0.21	0.27	0.16	0.16	0.34
Isolated	Precision	0.66	0.72	0.71	0.71	0.71
	Recall	0.57	0.84	0.88	0.88	0.62
	F1-Score	0.61	0.77	0.78	0.78	0.66
Death	Precision	0.08	0.71	0.56	0.56	0.30
	Recall	0.86	0.71	0.71	0.71	0.43
	F1-Score	0.15	0.71	0.63	0.63	0.35
	Accuracy	0.47	0.66	0.66	0.66	0.55

## Discussion

Machine learning techniques have been applied to the challenging problem of early prediction of mortality of intensive care unit (ICU) patients [37]. A patient’s healthcare utilization pattern may provide a more precise estimates of risk for adverse events (AE) or death [38]. To perform this prediction, a machine learning technique is used to predict the risk of AE or death within 90 days of surgery. In another study, electronic medical records (EMR) support the development of machine learning techniques for predicting disease incidence, patient response to treatment, and other healthcare events [39]. The machine learning model is used to optimize performance of predicting mortality and ICU stay time. Experiments reported in [40] showed that machine-learning approaches applied to raw electronic health records (EHR) data can be used to build models for use in research and medical practice. These approaches can identify novel predictive variables and their effects to inform future research in predicting patient mortality for coronary artery disease. The mortality rate of the novel 2019-nCoV continues to rise and we showed that machine learning techniques are useful for predictions in 2019-nCoV.

Our experiment showed vast improvement in prediction performance using ‘disease’ in the model. Such increase in the performance of these machine learning techniques is an indication of the high importance of including patient health information in 2019-nCoV cases. This will help clinicians to better predict the worst outcome for a 2019-nCoV patient. Using these predictions, better health-care measures can be targeted to those in need. This can result in a much higher increase in the number of ‘recovered’ cases. Additional datasets can strengthen these models in the future as more data become available. However, we note that even though 1.92% of the cases resulted in death for dataset2, AdaBoost was still able to obtain a significant recall value of 0.86, while bagging obtained a recall value of 0.71.

The AdaBoost ensemble model is used to classify and make accurate and reliable predictions for in-hospital mortality among patients with pancreatic cancer who undergo pancreatic resection [41]. In [42], bagging is one of the techniques used to predict if a United States heatwave is likely to result in high or moderate mortality. The bagging ensemble model performed admirably but further improvement was suggested. Another study [43] observed that in-hospital mortality of elective patients^1^ is low, because these admissions do not lead to an emergency or urgent admission. Nonetheless, there are still some cases of death for elective admission in hospitals. The researchers developed a technique by using machine learning-based models to predict death for the case of elective admissions. Bagging with the highest AUC can be considered to correspond to excellent discriminating performance. The AdaBoost and bagging models were effective in ‘death’ prediction for 2019-nCoV.

This result is spectacular and prompts immediate interest in the fruitfulness of using the bagging^2^ model built on sub-sample four in other 2019-nCoV datasets. At the time of writing this paper many more deaths have been reported than are used in these experiments. While the data for these deaths are not publicly available, our experiments showed that including ‘disease’ in datasets improves the performance of the models using machine learning techniques in ‘death’ prediction. This can be very valuable for clinicians in allocating treatment to 2019-nCoV patients. By utilizing either future datasets or the current dataset with additional data, the results obtained with this model can reduce the burden on health care systems worldwide.

Patients are quite uncertain whether they are diagnosed with 2019-nCoV and whether they really have this virus. Large health resources may be used to care for patients who in fact are not sick with 2019-nCoV but are still treated for this disease. This will consume valuable resources that can be allocated to patients who actually have 2019-nCoV. The results obtained from our system shows that there will be less chances of falsely predicting an 2019-nCoV. This means that health-care resources, for example hospitals could spend more time, staff effort and medical equipment including medicines on treating those cases that have 2019-nCoV. This could greatly lower their overall cost in treating with this virus. This the major reason for the focus of our study on obtaining good Recall values. This means that the predictions of the patients predicted to have 2019-nCoV are less likely to be incorrect. Consequently, using classifiers with good recall values will tremendously reduce the burden on health-care systems.

### Generalizations of the AUC for the multi-class setting

We determined multi-class AUC-ROC scores for each model in our experiments [44, 45]. Since AdaBoost and bagging were found to be the best models, we only present their multi-class AUC-ROC scores. In our first experiment, “Metrics of machine learning models for two most common outcomes on dataset1,” AdaBoost obtained 0.53, and bagging obtained 0.80. In our second experiment, “Metrics of machine learning models for three most common outcomes on dataset1,” AdaBoost obtained 0.68, while bagging obtained 0.66. In our third experiment, “Metrics of machine learning models for three most common outcomes on dataset2,” AdaBoost obtained 0.79, while bagging obtained 0.80. Last, in our final experiment, “Metrics of machine learning models for two most common and ‘disease’ outcomes on dataset2,” AdaBoost achieved 0.60, while bagging attained 0.74. For the purposes of this study, these multi-class AUC-ROC scores support our choice of bagging as the best classifier for death prediction.

### Binary classification ROC curves

**Fig. 4:**
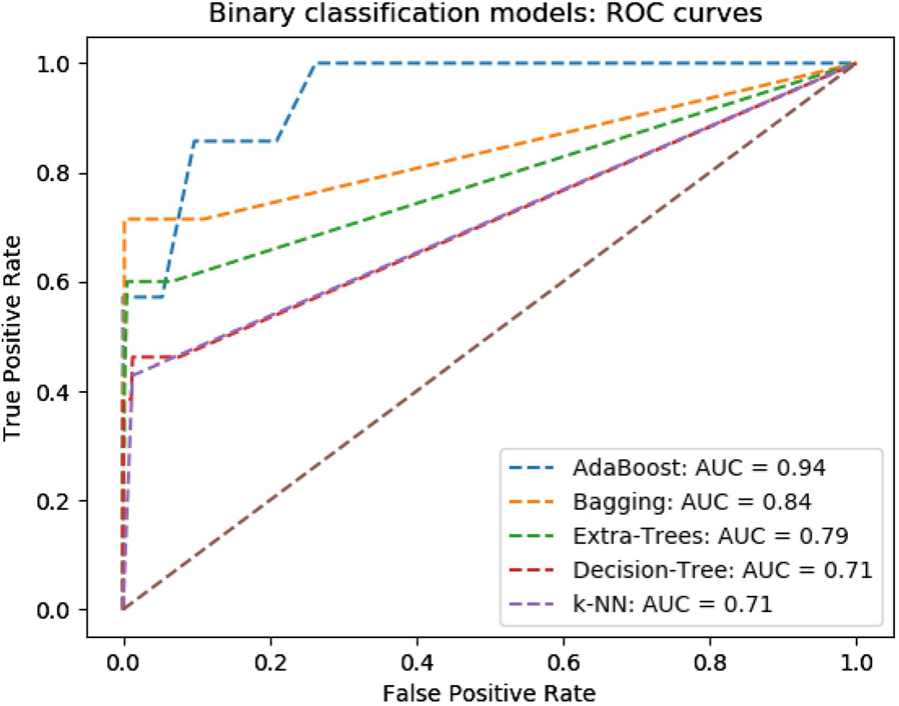
Binary classification models: ROC curves

Furthermore, we produced ROC curves [46] for all five binary classification models (Fig. 4) using our final experiment, "Metrics of machine learning models for two most common and 'disease' outcomes on dataset2". The curves were obtained using 'death' as the positive label, while all other labels were negative. The area under the ROC curve for AdaBoost binary classification was 0.94, while Bagging binary classification obtained an area of 0.84. This result supports the identification of AdaBoost and Bagging as good predictors for death prediction.

The ROC curves for Decision Tree and k-NN with an AUC of 0.71 were not significantly different from that obtained using Extra-Trees (AUC = 0.79). Also, the ROC curve for all the models are above the y = x line showing that it results in a high True Positive rate for the same False Positive rate. The AUC for AdaBoost is the highest followed by that of Bagging. Therefore, AdaBoost is the most appropriate classifier to predict the occurrence of Death.

### Hyper-parameter settings

Each experiment was run 1000 times with varying hyper-parameter value(s). The hyper-parameters were randomly chosen for each run. The best performance for each run based on our specified criteria. In addition, the hyper-parameter values were validated using Optunity [47, 48]. Even though we experimented on many different hyper-parameter settings for each model to attain an ‘optimal’ value, our attempts and the search performed using Optunity were not exhaustive. Thus, other researchers may be able to use hyper-parameter settings that may obtain better results than those in this study. However, because of the high computational overhead and time limits of achieving this possible outcome, these efforts are left for future work. For instance, for AdaBoost, the number of weak learners or estimators of 100 was experimentally found to be ‘optimal’ for this work but using other values with a tweaked learning rate may lead to more encouraging results. This may also apply to the bagging model that may be further optimized by varying the number of estimators and/or random state to values not generated in our experiments.

## Conclusion

This paper presents the results of using machine learning techniques to build models in order to predict 2019-nCoV deaths based on the patient demographics and health conditions. The AdaBoost and bagging machine learning models produced the best results in predictions ‘death’. These models demonstrate high predictive ability when trained with the disease feature. As additional data become available in the future, these models can be retrained to evaluate whether the model accuracy can be further improved. In addition, other features can be used to build new models using these machine learning techniques. This work should provide researchers with possible directions for developing further machine learning predictive models to help fight the 2019-nCoV outbreak. This can have a positive effect on predictive patient treatment and help ease the burden on the currently overloaded healthcare systems worldwide, especially with the increasing prevalence of second and third wave re-infections in some countries.

## Data Availability

Datasets obtained from Kaggle and listed in References section [no. 6 and 7]. Place here for reader convenience. SudalaiRajkumar: Novel Corona Virus 2019 Dataset. data retrieved March 30, 2020 from Kaggle, https://www.kaggle.com/sudalairajkumar/novel-corona-virus-2019-dataset (2020). KimHoo: Data Science for COVID-19 in South Korea. data retrieved March 30, 2020 from Kaggle, https://www.kaggle.com/kimjihoo/coronavirusdataset (2020).

## Abbreviations

nCoV: Novel coronavirus
RNA: Ribonucleic acid
k-NN: K-nearest neighbor
DT: Decision tree
NNC: Nearest neighbor classifier
TP: True positive
TN: True negative
FN: False negative
FP: False positive
CNN: Convolutional neural network
AI: Artificial intelligence
SVM: Support vector machine
RF: Random forest
MLPNN: Multilayer perceptron neural networks
UTC: Coordinated Universal Time
KCDC: Korea Centers for Disease Control & Prevention
ICU: Intensive care unit
AE: Adverse events
EMR: Electronic medical records
EHR: Electronic health records
ROC: Receiver operating characteristic
TPR: True positive rate
FPR: False positive rate
AUC: Area under the curve
AUC-ROC: Area under the ROC curve

## Footnotes

1 An elective procedure is one that is chosen (elected) by the patient or physician that is advantageous to the patient but is not urgent. Elective surgery is decided by the patient or their doctor.

2 Bagging has better general performance.
